# Effect of dietary supplementation with *Vitex negundo* L. var. cannabifolia extract on the growth performance, blood chemistry, gut morphology, and gut microbiota of broilers

**DOI:** 10.3389/fvets.2025.1713722

**Published:** 2025-11-24

**Authors:** Tianqi Huang, Xinggang Tang, Qianni Ye, Minggui Yuan, Xiaoai Zhang, Junheng He, Yali Cao, Qi Hu, Rong Xiang

**Affiliations:** 1 Institute of Animal Health, Guangdong Academy of Agricultural Sciences, Scientific Observation and Experiment Station of Veterinary Drugs and Diagnostic Techniques of Guangdong Province, Ministry of Agriculture and Rural Affairs, Guangzhou, China; 2 Key Laboratory of Livestock Disease Prevention of Guangdong Province, Guangdong Province Chinese Veterinary Medicine Engineering and Technology Research Center, Guangzhou, China; 3 Agro-Biological Gene Research Center, Guangdong Academy of Agricultural Sciences, Guangzhou, China

**Keywords:** *Vitex negundo* var. cannabifolia, broiler, growth performance, immunity, gut microbiome

## Abstract

The aim of this study is to evaluate the effects of *Vitex negundo* L. var. cannabifolia extract (VNE) on the growth performance, antioxidant status, blood chemistry, and cecal microbiota of broilers. A total of 240 one-day-old partridge broilers in total were randomly assigned to 5 treatment groups of 48 chicks each, which were divided into 6 replicates of 8 chicks. The first group (Control) was given the basal diet (only); the second group (Positive) was given the basal diet with 300 mg/kg of *Macleaya cordata* extract, and the low-dose group (Low), the middle-dose group (Mid) and the high-dose group (High) were given the basal diet with VNE at a dose of 1.3, 2.6 and 3.9 g/kg diet, respectively. The results showed significant improvements (*p* < 0.01) in growth performance, with significant improvements in body weight, weight gain, and feed conversion ratio at 3.9 g/kg diet. Broilers in the high-dose VNE group exhibited a significant reduction in serum total cholesterol (TC), alanine aminotransferase (ALT), and albumin (ALB) compared to the control group. Furthermore, this group showed a concurrent increase in immunoglobulins (IgA, IgG, and IgM). Furthermore, the morphology and microbial content of the jejunum and ileum were improved in broilers fed on a diet supplemented with a high dose of VNE compared to the control group. Cecal microbiome analysis showed that VNE addition obviously improved cecal microbial composition, as indicated by the increased relative abundance of Clostridia vadinBB60, the Rikenellaceae Rc9 gut_group, Christensenellaceae_R-7_group, Clostridia UCG-014, and Anaerofilum. In conclusion, dietary supplementation with VNE increased the productive performance, immunity, and blood chemistry profile, while modulating cecal microbiota in broiler chicks.

## 1 Introduction

Broiler chickens are a preferred choice in the poultry market due to their fast growth and exceptional feed efficiency, traits that are linked to their high metabolic energy demands (1). Various of low-inclusion feed additives, including phytogenics, acidifiers, prebiotics, probiotics, and antibiotics, have been added to broiler diets to enhance nutritional quality, improve animal performance, and promote health. Among these, antibiotics are commonly used specifically to mitigate the risk of bacterial infections (2, 3). However, excessive antibiotic use has led to bacterial resistance and environmental pollution, threatening animal welfare and reducing farm productivity (4). Thus, research on natural or synthetic alternatives to antimicrobials is growing, aimed at improving livestock health and maintaining regenerative production outcomes (5, 6). Among these, plant metabolites from traditional Chinese medicine have emerged as a key research focus owing to their wide availability, diverse sources, and antibacterial, antioxidant, and gut-regulating properties (7). For example, *Macleaya cordata* extract is a commercial natural feed additive used in poultry farming (e.g., chickens, ducks, and geese) via a multi-target regulation mechanism that improves growth and health performance (8). Additionally, *Scutellaria baicalensis* and *Lonicerae flos* extracts were found to promote growth performance and engage in inter-crosstalk with gut microbes to modulate gut barrier function (9). Therefore, it is a good opportunity to investigate more potential feed addictive among numerous natural herbs, and highlight further research to enhance our understanding of these herbs.

*Vitex negundo* L. var. cannabifolia (Sieb. & Zucc.) Hand.-Mazz. is a widely used herbal medicine in Traditional Chinese Medicine. It is documented in the Pharmacopoeia of the People’s Republic of China, with pharmacological effects including immunomodulatory, antipyretic, analgesic properties and anti-inflammatory properties (10, 11). Various chemical components, including lignans, flavonoids, terpenes, and phenolic acids, have been isolated from this plant and have been shown to exhibit aforementioned pharmacological activities (12, 13). However, few studies have investigated the use of *Vitex negundo* L. var. cannabifolia or its extracts in livestock production. Our previous study showed that the 60% ethanol extract of *Vitex negundo* L. var. cannabifolia has a high total flavonoid content (43.17 mg/g) and can improve growth performance in broiler chickens (14, 15). However, its active components and underlying mechanisms remain unclear.

This study tests the hypothesis that dietary supplementation with *Vitex negundo* L. var. cannabifolia extract (VNE) enhances broiler health and productivity by improving growth performance, modulating blood biochemistry, and promoting a beneficial cecal microbiota composition. Our findings aim to evaluate the potential of VNE as a sustainable phytogenic feed additive that could serve as an alternative to conventional growth promoters, thereby contributing to poultry production.

## 2 Materials and methods

### 2.1 Preparation of plant extract and phytochemical profiling

*Vitex negundo* L. var. cannabifolia were collected, dried, and subsequently pulverized with a grinder. Then, the whole plant powder was extracted with 30% EtOH for 3 h and freeze-dried to obtain the final extract.

A total of 0.8 mL of the filtered sample was prepared for detection by an H-Class ultraperformance liquid chromatography (UPLC) system (Waters, Massachusetts). Large-scale quantitative mass spectrometry using the Triple TOF 6600+ System (AB Sciex Framingham, Massachusetts) was used to analyze positive and negative ion modes. The detection system used an ACQUITY UPLC HSS T31.8 μm, 2.1 × 100 mm column. The mobile phase A was 0.1% formic acid in water, and the mobile phase B was 0.1% formic acid in acetonitrile. The elution rate was 0.4 mL/min, the column temperature was 40 °C, the collection time was 30 min, and the injection volume was 2 μL. The gradient elution procedure was as follows: 0–1.5 min at 0–5% B; 1.5–2.5 min at 5–10% B; 2.5–14 min at 10–40% B; 14–25 min at 40–95% B; 25–26 min at 95 to 5% B; 26–30 min at 5% B. To further determine the compound composition in VNE and eliminate redundant structures, the data were subjected to a database search and identification analysis. The AB Sciex mass spectrometry data were uploaded to Progenesis QI (Waters Technologies) and searched against a compound mass spectrometry database (a self-built database edited using MySQL, containing chemical structural formulas, names, mass spectrometry fragments, and so on, with the database file format being SDF). Compounds with a mass spectrometry fragment score >30 were then screened based on their matching scores.

### 2.2 Animals and experimental procedure

A total of 240 healthy and vaccinated one-day-old male partridge broilers (Tu No.5) were purchased from Guangdong Zhiwei Agricultural Technology Co., Ltd., with an average body weight of 53.51 ± 0.92 g. All experimental procedures were conducted in compliance with ethical guidelines and were approved by the Institutional Animal Care and Use Committee of the Institute of Animal Health at the Guangdong Academy of Agricultural Sciences, according to the Guangdong Province Laboratory Animal Management Regulations (YC-PT2024061). The birds were housed in galvanized metal cages (180 × 80 × 80 cm), with one feeder and one scaled water tank per cage. The experimental room was maintained at a constant temperature of 32 °C, and the relative humidity was maintained at 50–65%. A 23L:1D lighting schedule was used for the first 7 days, followed by a 20 L:4D schedule until euthanized. All equipment was cleaned and disinfected daily. No health abnormalities or mortality were observed, and all birds remained healthy for the duration of the study.

After a one-week acclimatization period, the broilers were randomly separated into 5 groups, with each group consisting of 6 replicates and 8 chickens per replicate: (1) the control group, (“Control”) which was fed a normal diet; (2) the positive group (“Positive”), which was fed a normal diet and 300 mg/kg of *Macleaya Cordata* extract, purchased from Hunan Micolta Biological Resources Co., Ltd. (Hunan, China); (3) the low dose group (“Low”), which was fed a normal diet and 1.3 g/kg of VNE; (4) the middle dose group (“Mid”), which was fed a normal diet and 2.6 g/kg of VNE; (5) the high dose group (“High”), which was fed a normal diet and 3.9 g/kg of VNE. The VNE supplementation doses (1.3, 2.6, and 3.9 g/kg of feed) were selected based on a prior pilot study. This range was chosen to establish a clear dose–response relationship and to deliver a targeted daily intake of flavonoids, which are the primary bioactive constituents of the extract. Throughout the 49-day experimental period, the broilers had free access to food and water ad libitum. Daily feed intake, along with the initial and final body weights, was recorded to evaluate average daily gain (ADG), average daily feed intake (ADFI), and the feed/gain ratio (F/G).

### 2.3 Sample collection

At 49 days of age, two birds per replicate were randomly selected from each of the 6 replicates per treatment group for a total of 12 birds per treatment group; they were fasted for 12 h and subsequently euthanized by cervical dislocation. After euthanization, blood samples were collected from the brachial vein under the wing and placed in pre-labeled 10 mL centrifuge tubes, tilted, and left to stand at 4 °C. After collection, the blood samples were centrifuged at 3000 rpm for 20 min at 4 °C. The supernatant serum was collected into 1.5 mL tubes and stored at −20 °C. After euthanization, the ceca were isolated and immediately transferred to clean tubes, and the cecal contents were collected from the cecum individually under a clean bench, immediately immersed in liquid nitrogen for initial preservation, and subsequently transferred to a −80 °C freezer for storage before the samples were sent to Majorbio (Majorbio Bio-Pharm Technology Co. Ltd., Shanghai, China) for microbial analysis.

### 2.4 Intestinal morphology

Immediately following euthanization, approximately 2–3 cm segments from the mid-jejunum and mid-ileum were collected. The segments were opened longitudinally, gently flushed with ice-cold phosphate-buffered saline (PBS, pH 7.4) to remove digesta, and then fixed in 4% paraformaldehyde solution for 24 h at 4 °C. After fixation, the tissues were dehydrated through a graded ethanol series, cleared in xylene, and embedded in paraffin wax. Serial sections of 5 μm thickness were cut using a microtome (Leica RM2235), mounted on glass slides, and stained with hematoxylin and eosin (H&E) following standard protocols. The stained sections were examined under a light microscope (Olympus CX23, Olympus, Japan) coupled with an image analysis system (cellSens Imaging, Olympus, Japan). Villus height (VH) was measured from the tip of the villus to the villus-crypt junction. Crypt depth (CD) was measured from the base of the crypt to the villus-crypt junction. For each intestinal segment per bird, 10 intact, well-oriented villi and their associated crypts were measured. The villus height to crypt depth ratio (VH: CD) was then calculated.

### 2.5 Analysis of clinical blood chemistry parameters

Twelve serum samples per treatment (for a total of 60 samples) were collected at 3,000 rpm for 20 min under 4 °C in a centrifuge. Direct Bilirubin (D-Bil), total Bilirubin (T-Bil), Triglycerides (TG), cholesterol (TC), Alkaline Phosphatase (ALP), Albumin (ALB), total protein (TP), alanine aminotransferase (ALT), and aspartate aminotransferase (AST) (Shenzhen Mindray Animal Medical Technology, China) were sent to the Automated Serum Chemistry Analyzer (Mindray BS 240 Measurement system, Shenzhen Mindray Animal Medical Technology, China) for analysis.

### 2.6 Quantification of cytokines by ELISA

Changes in the activities of serum total superoxide dismutase (T-SOD), Glutathione (GSH), and Malondialdehyde (MDA) were detected by ELISA according to the manual provided by the Nanjing Jiancheng Bioengineering Institute (Nanjing, China). Immune function analysis and inflammatory cytokine levels, including interleukin 1 beta (IL-1), interleukin 6 (IL-6), and tumor necrosis factor-alpha (TNF-α), were determined in serum using the immunoassay kits purchased from the Nanjing Jiancheng Bioengineering Institute (Nanjing, China).

### 2.7 Fecal 16S rRNA microbial analysis

Majorbio Bio-Pharm Technology Co., Ltd. (Shanghai, China) was entrusted to perform the 16S rRNA microbial analysis of broiler cecal contents. First, a DNA Kit was employed to isolate fecal DNA. Primers (forward: ACTCCTACGGGAGGCAG-CAG, reverse: GGACTACHVGGGTWTCTAAT) were used to amplify the 16S rRNA genes, and PCR reactions were performed in triplicate. Miseq libraries were prepared using the TruSeqTM DNA Sample Preparation Kit (Illumina, San Diego, CA) and were subsequently sequenced on the Illumina Miseq PE 300 platform (Majorbio Bio-Pharm Technology Co. Ltd., Shanghai, China). The sequencing data were analyzed on the Majorbio Cloud Platform.^1^ *α*-diversity was indicated by the Chao1, ace, Simpson, and Shannon indices, and *β*-diversity was calculated and compared by principal coordinate analysis (PCoA), which illustrated differences in bacterial composition among groups. Linear discriminant analysis effect size (LEfSe) was used to identify the enriched bacteria for each group, and only taxa meeting an LDA significance threshold of >4.0 were shown. *p*-values were calculated using the Wilcoxon test (see Table 1).

**Table 1:** Formulation and nutrient content of the basal diet (DM base).

Raw material	Content %	Content %
	7–21 days	22–49 days
Corn	50.91	54.53
Soybean meal	40.04	36.07
Soybean oil	4.88	5.74
Limestone	1	0.94
CaHPO4	2.18	1.97
Salt	0.45	0.34
Pre mix^a^	0.2	0.2
DL-methionine	0.24	0.13
Choline chloride (50%)	0.1	0.1
Total	100	100
Nutrients		
Metabolizable energy, Kcal/kg	3004.76	3,098
Protein, %	21.53	20
Calcium, %	0.98	0.9
Total phosphorus, %	0.73	0.8
Available phosphorus, %	0.44	0.4
Lysine, %	1.2	1.1
Methionine, %	0.56	0.43
Methionine + cysteine, %	0.91	0.76
Threonine, %	0.83	0.77
Tryptophane, %	0.26	0.24

a Each premix provides the following nutrients per kg of diet: vitamin A, 95 KIU; vitamin D_3_, 35 KIU; vitamin E, 180 mg; vitamin K_3_, 15 mg; vitamin B_2_, 50 mg; vitamin B_6_, 30 mg; vitamin B_5_, 17 mg; vitamin B_6_, 2.9 mg; vitamin B_12_, 50 mg; niacin, 300 mg; folate, 10 mg; zinc, 1,100 mg; iron, 1,000 mg; copper, 600 mg; iodine,1,100 mg; selenium,12 mg and cobalt, 100 mg.

### 2.8 Statistical analysis

All statistical analyses and graph generation were performed via SPSS 22.0 and GraphPad Prism 10.0. Production performance data were analyzed using a randomized design with pen as the experimental unit, and blood chemistry, serum ELISA, intestinal morphology, and cecal microbiome were analyzed using bird as the experimental unit. Comparisons involving three or more variables were analyzed using a one-way analysis of variance (ANOVA). For models showing a significant overall effect (*p* < 0.05), differences between individual treatment means were assessed using Tukey’s Honestly Significant Difference (HSD) post-hoc test. Correlations were assessed using Spearman’s rank-order correlation method. The data are presented as mean ± SEM. Significant differences are noted as follows: * (*p* < 0.05), ** (*p* < 0.01), and *** (*p* < 0.001).

## 3 Results

### 3.1 Comprehensive component spectrum identification

**Figure 1:**
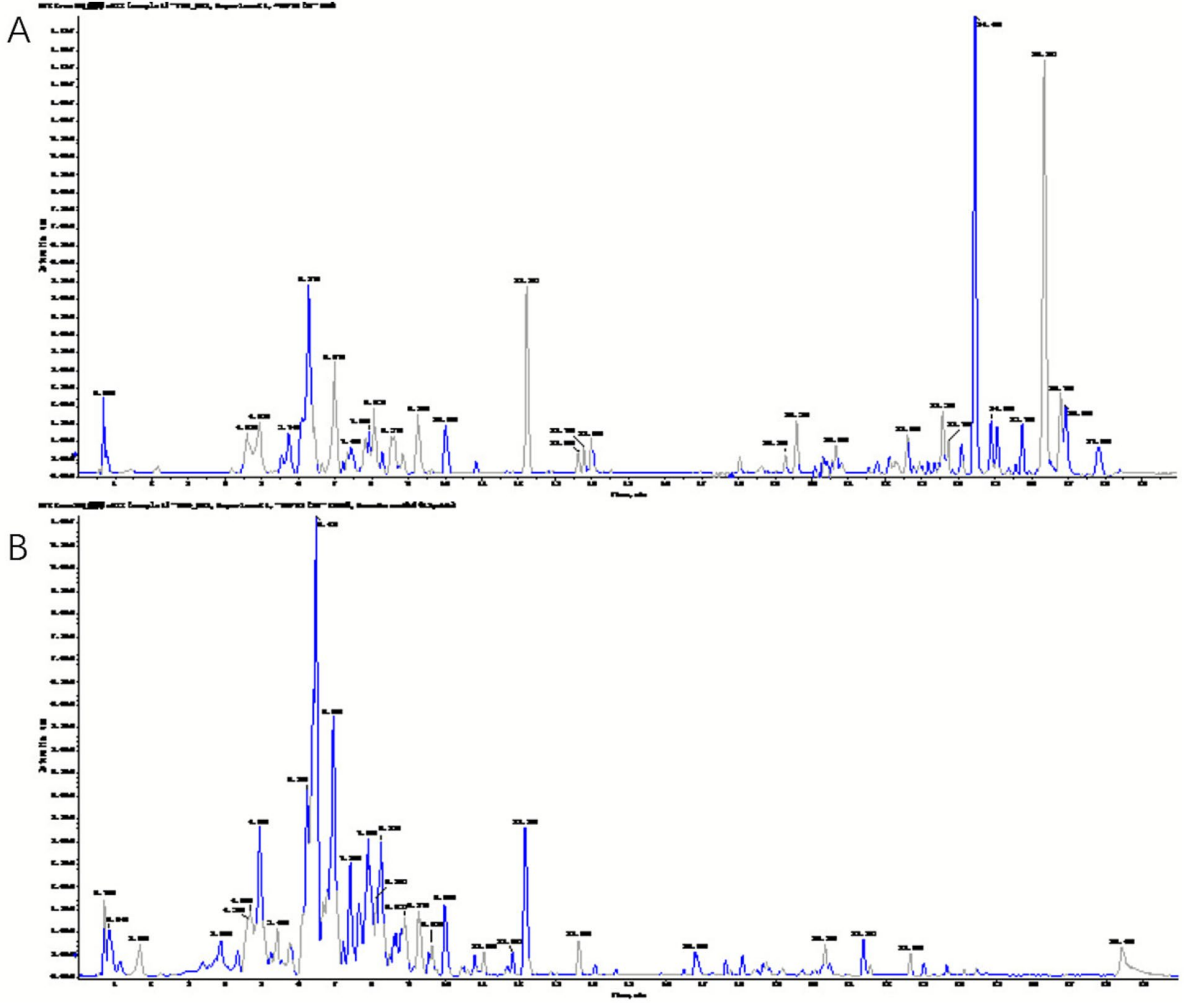
Components of *Vitex negundo* L. var. cannabifolia. The total ion chromatograms (*TIC*) for the positive **(A)** and negative **(B)** ion models.

The chemical components of VNE were analyzed via UPLC-TOF-MS. The total ion chromatograms (TICs) of the extracts in the positive and negative ion modes are shown in Figure 1. Based on a mass spectrometry fragment score threshold of >30, the ion fragments in the mass spectrometry results were compared with the MS/MS fragments in a compound mass spectrometry database and published literature. A total of 37 compounds were identified by searching the compound mass spectrometry database (Table 2). This diverse group of components included 28 flavonoids, 5 diterpenes, 2 lactones, and 2 phenolic acids.

**Table 2:** Identification results of *Vitex negundo* L. var. cannabifolia extract.

No.	Retention time	m/z	Compound	Adducts	Formula	Score	Mass error
1	1.22	185.0809	Aucubigenin	M+H-H2O, M+H	C9H12O4	51.4	−0.07
2	1.67	391.1252	Aucubin	M-H, M+FA-H	C15H22O9	45.3	−0.81
3	3.4	457.1134	Engeletin	M+Na	C21H22O10	31	6.54
4	3.88	375.1305	Mussaenosidic acid	M-H	C16H24O10	58.2	2.33
5	4.33	623.1646	Vitexin-2-O-rhamnoside	M+FA-H	C27H30O14	23.7	4.86
6	4.81	665.1761	Vitexin 2″-O-(4‴-O-acetyl) rhamnoside	M+FA-H	C29H32O15	18.4	6.11
7	5.25	341.123	Myzodendrone	M-H	C16H22O8	48.8	−3.59
8	6.47	465.1435	Agnuside	M-H	C22H26O11	46.2	6.92
9	6.64	567.1369	Iridin	M+FA-H	C24H26O13	22.5	2.61
10	7.02	431.1011	Apigenin 7-glucoside	M-H	C21H20O10	85.3	6.38
11	7.29	287.0561	Kaempferol	M+H	C15H10O6	56	3.97
12	7.84	543.2464	Agnucastoside B	M-H	C26H40O12	70.3	3.05
13	7.98	433.1137	Isovitexin	M+H	C21H20O10	66.2	1.9
14	8.27	447.096	Isoquercetin	M+H-H2O	C21H20O12	2.11	8.13
15	8.3	445.1117	4′-O-Methylvitexin	M-H	C22H22O10	25.1	−5.26
16	8.35	463.0898	Kaempferol-3-Arabofuranoside	M+FA-H	C20H18O10	55	3.93
17	8.57	341.1034	Belamcandin	M+H-H2O, M+H	C19H18O7	22.8	4.08
18	8.94	433.1133	Vitexin	M+H	C21H20O10	61.9	0.81
19	9.18	525.2319	Agnucastoside A	M+H-H2O	C26H38O12	26.2	−2.12
20	10.04	285.041	5,7,2′,3′-Tetrahydroxyflavone	M-H	C15H10O6	34.9	1.91
21	10.06	301.0356	Quercetin	M-H2O-H, M-H	C15H10O7	56.1	−4.69
22	10.13	553.1353	Luteolin 7-(6″-p-benzoyglucoside)	M+H	C28H24O12	37.2	2.3
23	10.43	683.2031	Agnucastoside C	M-H	C34H36O15	54.9	7.24
24	10.84	579.1515	Vitexin2″-O-p-coumarate	M+H-H2O, M+H, M+Na	C30H26O12	64.4	3.05
25	12.23	361.0939	Irigenin	M+H, M+Na	C18H16O8	37.3	5.9
26	12.47	341.103	Penduletin 4′-methyl ether	M+H-H2O	C19H18O7	20.1	2.89
27	12.92	329.2342	Sanleng acid	M-H	C18H34O5	30.5	2.56
28	13	315.0524	Apigenin	M+FA-H	C15H10O5	14.3	5.09
29	14.31	375.1086	Casticin	M+H, M+Na	C19H18O8	35.1	3.11
30	16.49	393.23	Viteagnusin I	M-H	C22H34O6	90.1	4.49
31	16.79	487.3454	Lycoclavanin	M-H, M+FA-H	C30H48O5	56.9	5.19
32	18.09	423.2415	Vitexilactone	M-H, M+FA-H	C22H34O5	33.6	2.54
33	20.27	409.3464	β-sitosterol	M+Na	C27H46O	51.1	5.92
34	23.01	439.3588	Betulinic acid	M+H-H2O, M+H	C30H48O3	41.2	3.88
35	24.11	349.2743	Viteagnusin A	M+H-H2O, M+H	C22H36O3	35.6	1.04
36	24.11	291.2686	Viteagnusin C	M+H-H2O, M+H	C20H36O2	80.8	1.14
37	27.79	397.3838	β-sitosterol	M+H-H2O	C29H50O	36.9	2.24

### 3.2 Effect of VNE supplementation on growth performance

The feeding regimen consisted of two phases: an early (7–21 days) and a late (21–49 days) to evaluate changes in dietary response. During the early phase, there were no significant differences in growth performance between the Control and VNE-treated groups. However, obvious changes in ADG and FCR occurred during the late phase (*p* < 0.05; Table 3). Interestingly, ADG increased considerably (*p* < 0.001) in all VNE-treated groups compared to the control group. By the 49th day of the experiment, the difference in ADG between the VNE-treated groups and the control group was more pronounced, while the ADG of broilers in the low group (*p* < 0.01) was significantly higher than that of the control group, and the mid (*p* < 0.001) and high (*p* < 0.001) groups showed a significantly higher ADG than the control group. As shown in Table 3, the FCR of the low group (*p* < 0.01), medium group (*p* < 0.001), and high group (*p* < 0.001) showed a highly significant decrease compared to the control group.

**Table 3:** Effect of VNE supplementation on the growth performance of broilers^1^.

Age (day)	Item^2^	Ctrl	Positive	Low	Mid	High	*P-*value
7–21 days	ADFI	37.07 ± 0.023	36.61 ± 0.125	36.32 ± 0.366	36.03 ± 1.208	35.95 ± 0.554	0.155
	ADG	16.43 ± 0.222	16.26 ± 0.213	17.11 ± 0.251	16.93 ± 0.208	16.71 ± 0.234	0.063
	FCR	2.37 ± 0.088	2.25 ± 0.134	2.12 ± 0.066	2.12 ± 0.087	2.15 ± 0.114	0.326
21–49 days	ADFI	67.16 ± 0.991	67.07 ± 0.416	65.42 ± 1.332	65.38 ± 0.202	65.80 ± 0.011	0.099
	ADG	28.23 ± 0.687^b^	30.09 ± 0.648^b^	31.73 ± 0.705^a^	32.17 ± 1.189^a^	32.35 ± 1.243^a^	0.036
	FCR	2.45 ± 0.081^b^	2.23 ± 0.101^b^	2.07 ± 0.015^a^	2.03 ± 0.016^a^	2.03 ± 0.191^a^	0.022
7–49 days	ADFI	57.11 ± 0.111	56.92 ± 0.591	55.72 ± 0.567	55.60 ± 0217	55.86 ± 0.462	0.11
	ADG	24.61 ± 0.621^c^	25.48 ± 0.589^c^	26.86 ± 1.68^b^	26.88 ± 0.651a	27.14 ± 0.153^a^	<0.001
	FCR	2.37 ± 0.038^c^	2.23 ± 0.054^c^	2.07 ± 0.033^b^	2.07 ± 0.073^a^	2.06 ± 0.191^a^	<0.001

^1^Means (*n* = 6) within a row with different letters were significantly different (*p* < 0.05).

^2^ADFI, average daily feed intake; ADG, average daily weight gain; FCR, feed conversion ratio.

### 3.3 Effect of VNE supplementation on the intestinal morphology of broilers

**Figure 2:**
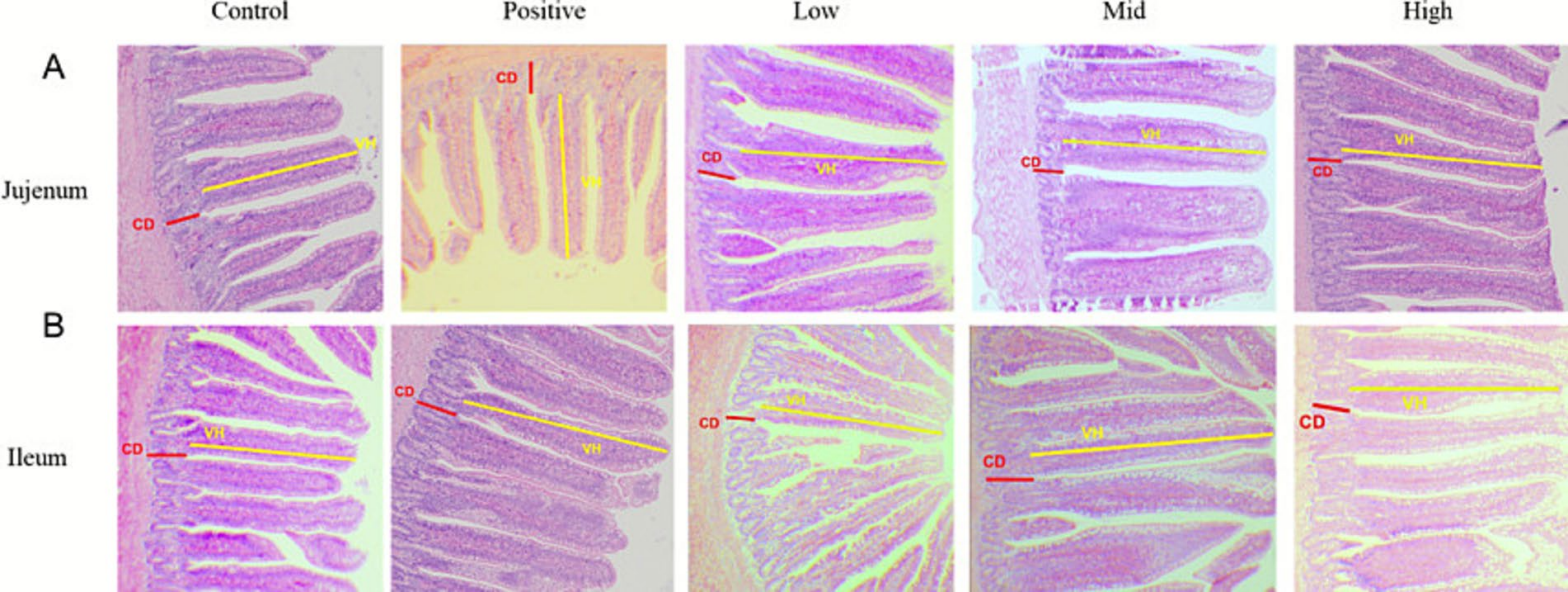
Effect of VNE supplementation on the intestine morphology of broilers. Representative images of the jejunum **(A)** and ileum **(B)** morphological structure, which was observed at 40× magnification.

The effect of dietary VNE supplementation on the jejunal and ileal histomorphometry of 49-day-old broilers is shown in Figure 2. There were no significant changes in the villus height (VH) or crypt depth (CD) (*p* > 0.05) between the low-dose and high-dose groups. As shown in Table 4, jejunal VH increased significantly (*p* < 0.01) in the VNE-treated group, and ileal villus height increased significantly (*p* < 0.001) in the high-dose group. The villus-to-crypt ratio increased significantly in the low-dose group and high-dose groups for the jejunum and ileum. The VH/CD ratio increased significantly in the low- and high-dose groups for the jejunum (*p* < 0.01) and ileum (*p* < 0.05).

**Table 4:** Effect of VNE supplementation on the intestine morphology of broilers^1^.

Item	Control	Positive	Low	Mid	High	*p-*value
Jejunum						
Villi height/μm	799.32 ± 16.613^b^	894.01 ± 22.508^b^	934.95 ± 19.393^a^	908.87 ± 17.262^a^	939.31 ± 22.601^a^	<0.001
Crypt depth/μm	56.94 ± 2.065	56.91 ± 2.455	55.73 ± 2.943	58.26 ± 2.641	56.05 ± 2.897	0.12
VH/CD	14.16 ± 0.411^c^	16.13 ± 1.101^b^	17.27 ± 1.011^a^	15.95 ± 0.835	18.05 ± 0.501^a^	0.019
Ileum						
Villi height/μm	511.98 ± 1.671^c^	540.78 ± 1.268^b^	567.67 ± 9.475^b^	568.28 ± 14.815^b^	586.29 ± 10.831^a^	<0.001
Crypt depth/μm	47.63 ± 1.363	48.18 ± 1.655	46.87 ± 1.518	45.94 ± 1.412	47.88 ± 1.567	0.359
VH/CD	10.84 ± 0.334^c^	11.51 ± 0.401^b^	12.6 ± 0.466^a^	11.72 ± 0.399^b^	12.42 ± 0.409^a^	0.038

^1^Means (*n* = 12) within a row with different letters were significantly different (*p* < 0.05).

### 3.4 Effect of VNE supplementation on the clinical blood chemistry of broilers

The results in Table 5 show that VNE treatment slightly reduced the levels of direct bilirubin (D-Bil), total bilirubin (T-Bil), and aspartate aminotransferase (AST) in the serum of 49-day-old broilers (*p* > 0.05). Additionally, the serum alanine aminotransferase (ALT) concentration significantly decreased in both the low-dose and high-dose groups (*p* < 0.05). The alkaline phosphatase (ALP) concentration increased in the high-dose VNE treatment group, while both dietary treatments, the phytogenic positive control and the VNE, led to a significant reduction in serum triglyceride (TG) (*p* < 0.05) levels compared to the control group (Table 5), suggesting an improvement in lipid metabolism. Serum albumin (ALB) levels were significantly elevated in the low-dose and high-dose groups (*p* < 0.05). Notably, the triglyceride content significantly decreased in the positive control and the Low groups (*p* < 0.05), with a more pronounced reduction in the Mid-and High groups (*p* < 0.01 and *p* < 0.001, respectively). Although the serum total cholesterol (TC) content showed a decreasing trend, the difference did not reach statistical significance.

**Table 5:** Effect of VNE supplementation on the blood biochemistry of broilers^1^.

Item^2^	Control	Positive	Low	Mid	High	*p*-value
D-Bil (μmol/L)	1.19 ± 0.063^b^	1.17 ± 0.116^b^	1.04 ± 0.109^b^	0.99 ± 0.093*	0.88 ± 0.129^b^	0.053
T-Bil (μmol/L)	2.75 ± 0.113	2.76 ± 0.127	2.44 ± 0.164	2.47 ± 0.098	2.42 ± 0.204	0.344
ALT (U/L)	1.909 ± 0.426	2.172 ± 0.261	1.168 ± 0.288	1.708 ± 0.241	1.434 ± 0.288	0.317
AST (U/L)	218.38 ± 20.351^b^	211.13 ± 9.248^b^	216.61 ± 12.075^b^	210.21 ± 7.974^b^	204.81 ± 7.481^a^	0.024
ALP (U/L)	1752.11 ± 153.55^b^	1721.1 ± 173.143^b^	1760.24 ± 194.769^b^	1706.34 ± 135.175^b^	1291.57 ± 127.406^a^	0.007
TP (g/L)	24.64 ± 3.967^b^	33.34 ± 0.773^a^	38.21 ± 2.393^a^	35.56 ± 1.255^a^	35.84 ± 2.611^a^	<0.001
ALB (g/L)	10.34 ± 0.509	12.14 ± 0.324^b^	13.06 ± 0.848^b^	12.14 ± 0.515^b^	12.6 ± 1.039^a^	0.015
TG (mmol/L)	0.58 ± 0.103^b^	0.37 ± 0.041^a^	0.39 ± 0.04^a^	0.35 ± 0.029^a^	0.29 ± 0.019^a^	0.032
TC (mmol/L)	3.06 ± 0.308^b^	3.85 ± 0.161^a^	3.79 ± 0.017^a^	3.45 ± 0.145^a^	3.38 ± 0.14^a^	0.043

^1^Means (*n* = 12) within a row with different letters were significantly different.

^2^D-Bil, Direct Bilirubin; T-Bil, Total Bilirubin; ALT, alanine transaminase; AST, aspartate transaminase; ALP, Alkaline Phosphatase; TP, Total Protein; ALB, Albumin; TG, Triglycerides; TC, Total cholesterol.

### 3.5 Effect of VNE supplementation on cytokine release of broilers

As shown in Table 6, serum malondialdehyde (MDA) activity decreased significantly in the VNE-treated groups compared with the control group (*p* < 0.01). Superoxide dismutase (SOD) activity increased significantly (*p* < 0.01) in both the low- and high-dose groups, while glutathione (GSH) levels were significantly higher in all the VNE-treated groups (*p* < 0.05). Following VNE supplementation, the production of interleukin-1β (IL-1β), interleukin-6 (IL-6), and tumor necrosis factor-α (TNF-α) decreased in a dose-dependent manner (*p* < 0.05).

**Table 6:** Effect of VNE supplementation on the serum cytokine of broilers^1^.

Item^2^	Control	Positive	Low	Mid	High	*p*-value
MDA (mmol/L)	35.68 ± 3.82^b^	30.63 ± 5.79^b^	23.32 ± 3.44^a^	29.23 ± 3.93^b^	28.09 ± 0.54^a^	0.009
SOD (U/mL)	39.24 ± 4.342^b^	69.53 ± 10.85^a^	67.89 ± 4.60^a^	65.59 ± 5.071^a^	68.80 ± 3.33^a^	0.009
GSH (μmol/L)	7.24 ± 0.98^c^	13.14 ± 1.98^a^	15.89 ± 1.32^a^	10.98 ± 1.713^b^	12.40 ± 2.75^b^	0.007
IL-1β (ng/mL)	11.87 ± 1.71^c^	7.23 ± 0.82^b^	6.75 ± 0.83^b^	7.46 ± 0.979^b^	3.5 ± 0.639^a^	<0.001
IL-6 (ng/mL)	12.81 ± 0.81^b^	10.99 ± 3.12^a^	5.07 ± 1.53^a^	7.65 ± 2.771^a^	4.87 ± 0.53^a^	<0.001
TNF-a (ng/mL)	503.24 ± 32.68^c^	358.34 ± 28.93^b^	388.69 ± 20.04^b^	354.97 ± 35.05^b^	324.2 ± 30.47^a^	0.004
IgA (ng/mL)	178.33 ± 3.51^b^	189.93 ± 13.66^b^	179.48 ± 13.66^b^	220.43 ± 17.24^b^	262.83 ± 6.59^a^	<0.001
IgG (ng/mL)	414.99 ± 40.98^b^	589.8 ± 53.35^a^	458.46 ± 29.32^b^	561.29 ± 34.47^a^	628.51 ± 57.55^a^	0.014
IgM (ng/mL)	259.23 ± 15.47^b^	273.69 ± 23.35^b^	312.66 ± 21.09^a^	371.76 ± 15.71^a^	383.47 ± 31.06^a^	0.002
IGF-1 (ng/mL)	271.08 ± 18.01^b^	354.83 ± 51.73^a^	408.72 ± 28.05^a^	405.83 ± 23.21^a^	417.09 ± 40.17^a^	0.037
GH (ng/mL)	27.35 ± 2.63^b^	59.42 ± 11.91^a^	58.18 ± 8.18^a^	63.62 ± 8.78^a^	60.81 ± 4.74^a^	0.021

^1^Means (*n* = 12) within a row with different letters were significantly different.

^2^MDA, Malondialdehyde; SOD, superoxide dismutase; GSH, Glutathione; IL-1β, interleukin 1β; IL-6, interleukin 6; TNF-α, tumor necrosis factor alpha; IgG, Immunoglobulin G; IgA, Immunoglobulin A; IgM, Immunoglobulin M; IGF-1, Insulin-like Growth Factor-1; GH, growth hormone.

The effect of dietary VNE supplementation on immunoglobulins in broilers was also evaluated. Broilers fed with the VNE-supplemented diet exhibited significantly higher IgG levels than the control group (*p* < 0.05). IgM and IgA levels were also significantly affected (*p* < 0.05). Furthermore, insulin-like growth factor-1 (IGF-1) levels were significantly higher in all VNE treatment groups than in the control group (*p* < 0.05). Growth hormone concentrations also increased significantly in all VNE treatment groups (*p* < 0.05, Table 6).

### 3.6 Analysis of the cecal microbiota community by 16S rRNA sequencing

**Figure 3:**
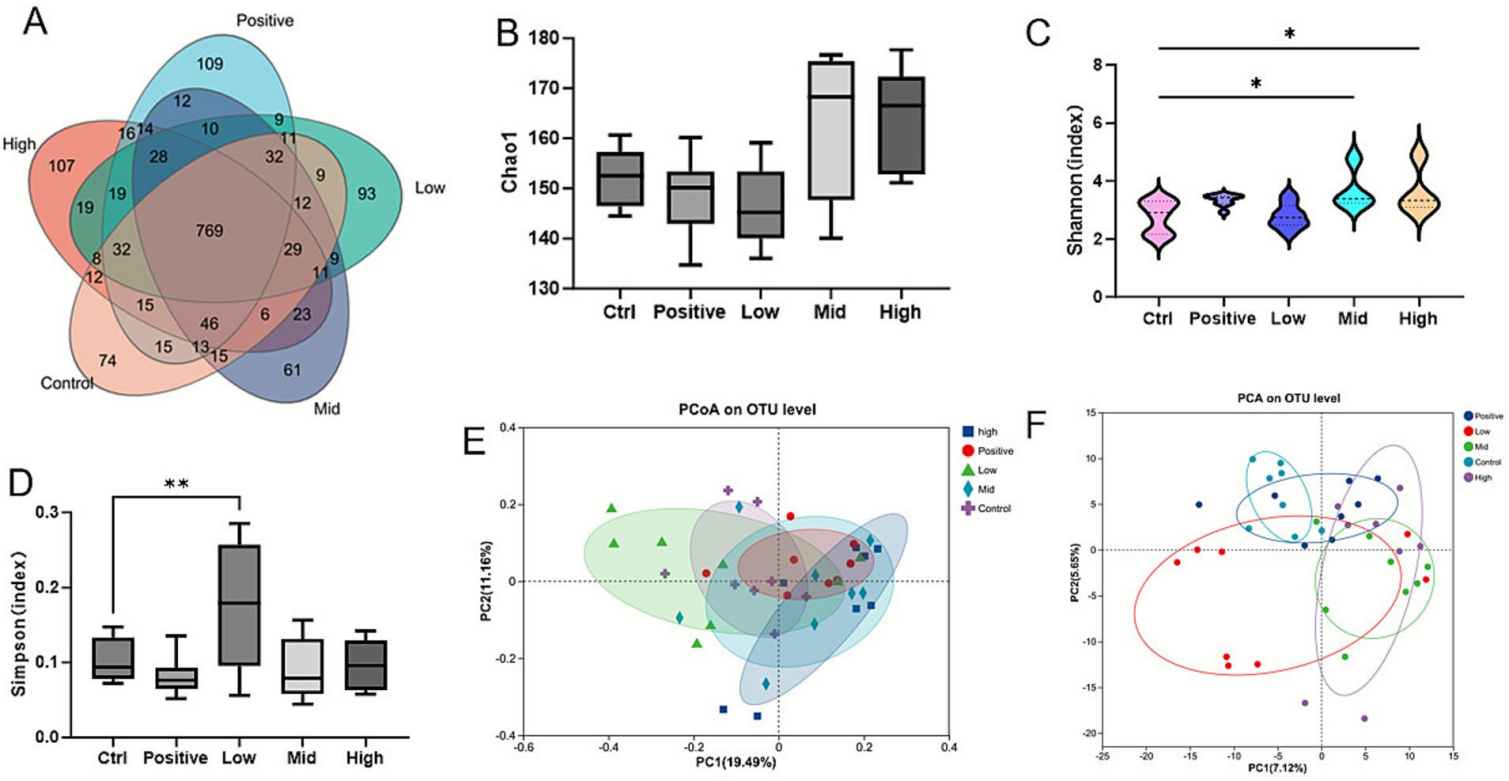
Effect of VNE supplementation on the diversity of the cecal microbiota of broilers. **(A)** A Venn diagram based on the OTU level. Alpha diversity indices observed species **(B)**, Chao1 index, **(C)** Shannon index, **(D)** Simpson index. **(E)** Principal coordinate analysis (PCoA) based on Bray-Curtis. **(F)** Principal components analysis (PCA) based on Bray-Curtis. Compared with the control group, values with significant differences were marked (*n* = 12; *: *p* < 0.05, **: *p* < 0.01).

To investigate changes in the composition and structure of the gut microbiota among all broilers, 16S rRNA sequencing was performed on the cecal contents. A total of 1,638 operational taxonomic units (OTUs) were detected across the five treatment groups, 769 of which were shared among all treatments. The control group contained 74 unique OTUs, while the positive, low-dose, mid-dose, and high-dose VNE groups had 109, 93, 61, and 107 unique OTUs, respectively (Figure 3A).

**Figure 4:**
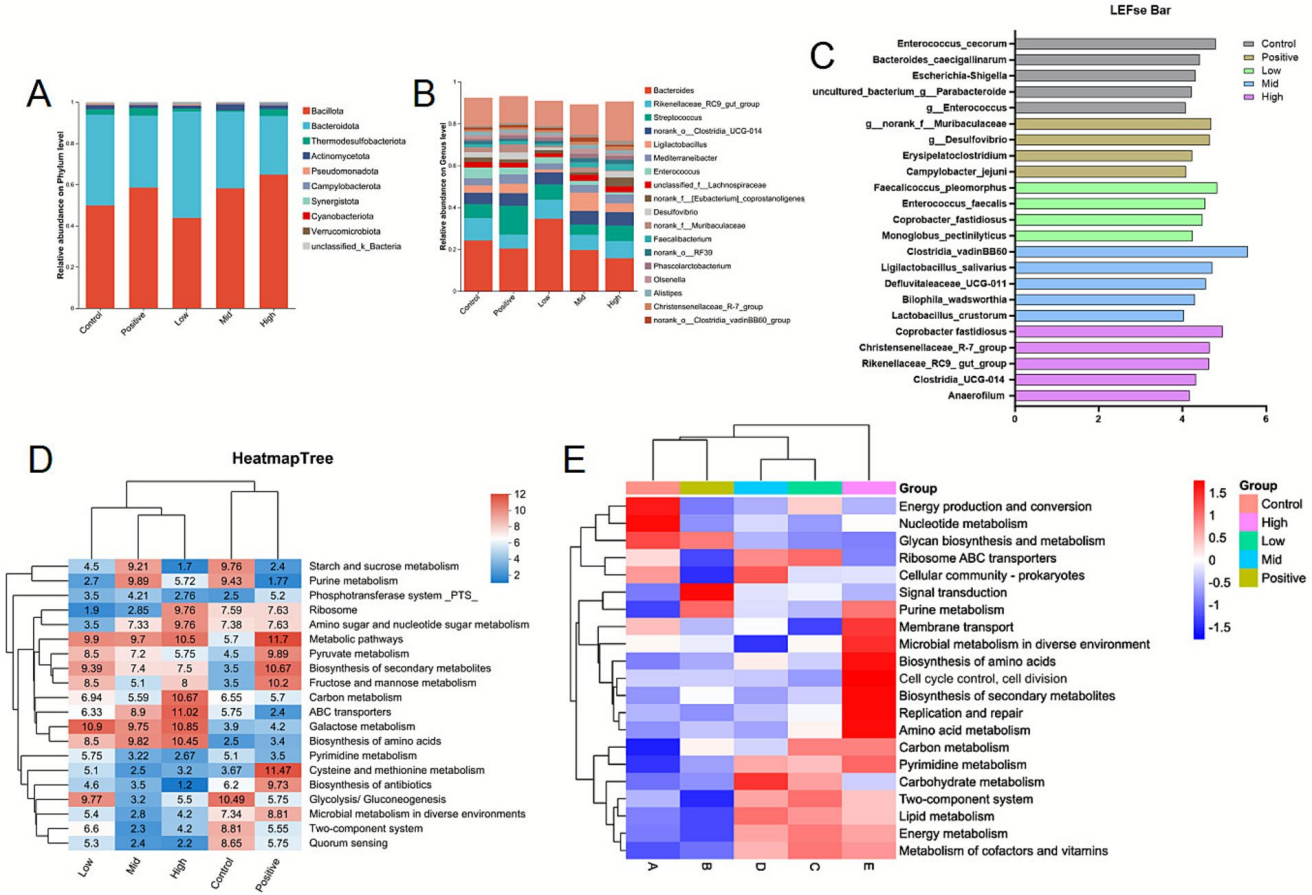
Effect of dietary supplementation with VNE on the cecal microbiota of broilers. **(A)** Microbial composition at the phylum level; **(B)** microbial composition at the genus level; **(C)** linear discriminant analysis effect (LEfSe) size of the intestinal microbiota (LDA > 4, *p* < 0.05); **(D)** predicted functional categories annotated by KEGG at Level 2 using Tax4Fun2; **(E)** predicted functional categories annotated by KEGG at Level 2 using PICRUSt2.

The results of the cecal microbiota analysis showed no significant differences in the observed species or Chao1 index (*p* > 0.05; Figure. 3B). However, the Shannon index tended to increase (*p* < 0.05; Figure. 3C) in the mid and high-dose groups, and the Simpson index increased in the low-dose group (*p* < 0.01; Figure 3D) following VNE supplementation. Principal coordinate analysis (PCoA) and principal component analysis (PCA) showed that the microbial composition of the cecal digesta of broilers in the high-dose group was distinctly separate from that of the control group (Figures 3E,F). The top 10 phyla and 20 genera are highlighted in Figures 4A,B. At the phylum level, the dominant bacteria (relative abundance ≥5.0%) in the control group were Bacillota and Bacteroidota. Supplementation with AGP alternatives or VNE altered the cecal microbial community structure (Figure 4A). In the mid and high-dose groups, Bacillota was more dominant than in the control group, and a similar increase was also observed in the positive group. At the genus level, the dominant bacteria included Bacteroides, the Rikenellaceae Rc9 gut group, *Streptococcus*, *Clostridia* UCG-014, and Ligilactobacillus (Figure 4B).

Furthermore, LEfSe analysis was performed to identify taxa with significantly different relative abundances among groups. As shown in Figure 4C, VNE supplementation altered the distribution of differentially abundant taxa in OTUs between the control and VNE-treated groups. At the genus level, the dominant genera in the control group were *Enterococcus cecorum*, Bacteroides caecigallinarum, Escherichia shigellaun, an uncultured bacterium_g Parabacteroide, and g_Enterococcus, whereas in the high-dose group, Coprobacter fastidiosus, Christensenellaceae_R-7_group, Rikenellaceae Rc9_gut_group, Clostridia_UCG-014, and Anaerofilum were more abundant. Overall, these findings suggest that VNE supplementation modifies the diversity and composition of the intestinal microbiota of broilers.

To further explore the metabolic potential of the altered microbial communities, functional and phenotypic abundance analyses were conducted. The Tax4Fun2 functional abundance analysis (Figure 4D) indicated that these microorganisms were associated with various pathways, including cell cycle control, cell division, microbial metabolism in diverse environments, two-component systems, pyrimidine metabolism, carbohydrate metabolism, lipid metabolism, cofactor and vitamin metabolism, and energy metabolism. Additionally, the PICRUSt2 functional abundance analysis revealed the involvement of these microorganisms in carbon metabolism, biosynthesis of secondary metabolites, biosynthesis of amino acids, amino acid metabolism, urine metabolism, cell cycle control, cell division, microbial metabolism in diverse environments, replication and repair, pyrimidine metabolism, carbohydrate metabolism, and lipid metabolism (Figure 4E).

### 3.7 Correlation analysis

**Figure 5:**
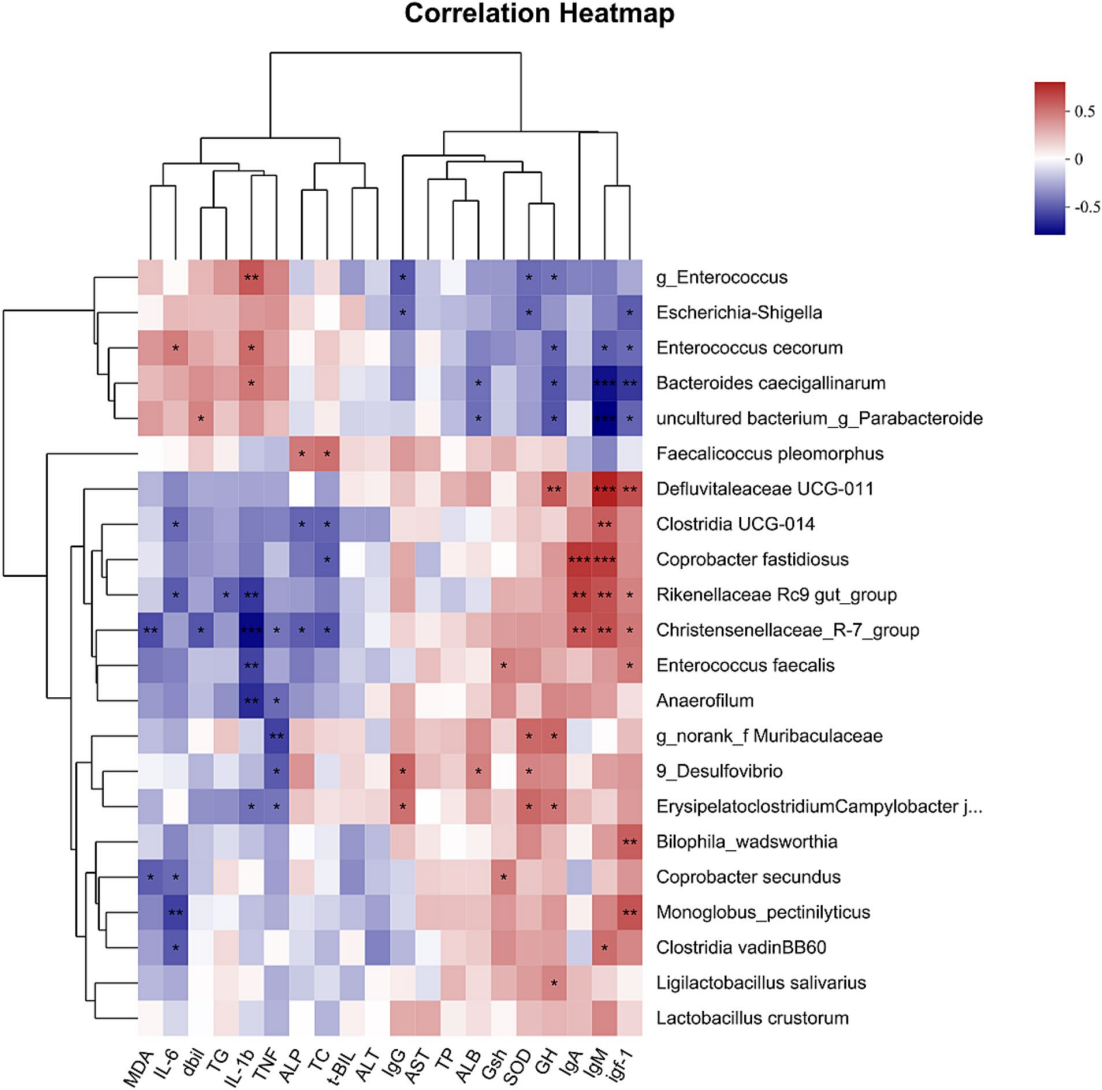
Correlation analysis between the gut microbiome and the serum development-related index. Heat map of Spearman’s correlation coefficient between the gut microbiota and the serum development-related index. In the figure, blue indicates negative correlations, and red represents positive correlations. *: *p* < 0.05, **: *p* < 0.01, ***: *p* < 0.001. Data are shown as means ± SEM; *n* = 12.

Spearman correlation analysis was performed to examine the relationship between gut microbiota and intestinal development-related indices. The abundance of Christensenellaceae_R-7_group, Rikenellaceae Rc9_gut_group, and *Coprobacter fastidiosus* was positively correlated with the IgM and IgA levels (*p* < 0.001), while the abundance of Christensenellaceae_R-7_group and Rikenellaceae Rc9_gut_group was negatively correlated with the IL-1β level (*p* < 0.01). Bacteroides caecigallinarum and an uncultured bacterium g Parabacteroides, were negatively related to IgM and IgA levels (*p* < 0.01) in addition to IGF-1 levels (*p* < 0.05), while positively related to IL-1β levels (*p* < 0.01). Moreover, Christensenellaceae_R-7_group was negatively correlated with T-Bil, TC, ALP, and D-Bil (*p* < 0.05), while it was also negatively correlated with MDA level abundance (*p* < 0.01) (see Figure 5).

## 4 Discussion

Our study demonstrated that dietary supplementation with VNE enhanced growth performance in broilers, suggesting that VNE positively influences body weight gain and feed conversion ratio (FCR). The main advantage of plant extracts is their rich composition of bioactive compounds, which enables them to produce unique effects via multiple mechanisms. *Vitex negundo* L. var. Cannabifolia is a potential feed supplement for the livestock industry because it contains valuable bioactive ingredients, such as flavonoids and terpenoids (16). In this study, we expected that adding VNE to the diet would improve growth performance in broiler chickens. It is well recognized that natural products improve broiler growth performance by modulating a healthy intestinal environment (17). Consistent with this, the enhancement in broiler growth performance following VNE treatment was closely associated with improved gut function, as evidenced by beneficial changes in intestinal morphology and strengthened mucosal barrier integrity. Intestinal morphology, including villus height, crypt depth, and the villus-to-crypt depth ratio, plays a crucial role in nutrient digestion and absorption. In the present study, VNE supplementation increased jejunal and villus height, in addition to the villus height-to-crypt depth ratio, indicating an expansion of the intestinal absorptive surface area and establishing an optimized microenvironment for efficient nutrient uptake (18).

While the present study demonstrates the efficacy of VNE, a balanced discussion requires consideration of its safety profile. It is important to note that the bioactivity of phytogenic compounds is inherently dose-dependent, where sub-therapeutic levels may be ineffective and excessively high doses could potentially induce adverse effects (19). A critical finding of the present study is that no mortality or abnormal development was observed in broilers fed VNE at any supplementation level (up to 3.9 g/kg). This, combined with the absence of negative impacts on blood chemistry and intestinal function reported in the results, provides strong *in vivo* evidence that the dosage range used here falls within a safe and therapeutic window.

Biochemical indicators of liver and kidney function, along with blood chemistry, offer valuable insight into the overall health and physiological state of broilers (20). In our study, the VNE-supplemented diet resulted in significant differences in blood serum concentrations and liver enzyme activities. Elevated ALT levels are commonly associated with liver damage or dysfunction (21), while reduced levels are often indicative of improved liver health (22). The decrease in ALT levels observed in our study suggests that VNE treatment may enhance liver function, likely due to its antioxidant and anti-inflammatory effects. Total protein (TP) and albumin levels may reflect protein metabolism and hepatic status (23). Enhanced TP and ALB levels are indicators of hepatic function, and supporting hepatic function in broilers by supplementing their diets with VNE may help protect their livers from damage. Triglycerides (TG) are the main constituents of body fat in animals, and total cholesterol (TC) is an important constituent (30%) of all cell membranes. Research in both humans and animal models has established that elevated serum triglycerides (TG) and total cholesterol (TC) are independent risk factors for the development of cardiovascular and hepatic metabolic disorders, which are often driven by lipid accumulation and oxidative stress (24, 25). In the present study, dietary supplementation with VNE significantly reduced serum TG concentrations and induced a downward trend in TC levels. This hypolipidemic effect suggests that VNE actively contributes to maintaining systemic lipid homeostasis in broilers. The observed reduction is likely attributable to VNE’s rich flavonoid content, which may inhibit hepatic *de novo* lipogenesis and/or enhance fatty acid β-oxidation. By promoting a healthier blood lipid profile, VNE supplementation could play a crucial role in protecting broilers from metabolic ailments associated with lipid dysregulation, thereby supporting overall metabolic health.

Plant medicines have been reported to exhibit favorable antioxidant properties in animals (26). The primary defense mechanism of antioxidant systems involves the suppression of free radical generation and lipid peroxidation, both of which elevate MDA production. Free radical-induced cell damage leads to lipid peroxidation, the extent of which can be quantified by measuring Malondialdehyde (MDA), a key end product of this process (27). The results indicate that adding VNE to the diets slightly decreased serum MDA concentration in broilers. Additionally, there was a marked increase in SOD and GSH activities in the serum of broilers fed with VNE. These results reveal that the inclusion of VNE in feed could alleviate oxidative stress in chickens by enhancing their endogenous antioxidant defense system. Consistent with this, the use of other flavonoid-based probiotics also resulted in elevated SOD and GSH activities and reduced MDA concentrations in broilers (28). Plant-based probiotics can regulate the immune response by stimulating cytokines, which in turn stimulate host resistance and promote the elimination of potential pathogens (29). Inflammatory factors include IL-1β, IL-6, and TNF-α. These factors are important inflammatory mediators in animals and play significant roles in immune regulation. In this study, VNE supplementation reduced the secretion of IL-1β, IL-6 and TNF-α, which helped chickens protect themselves from harmful physical or chemical stimuli, decreased the level of IL-1, promoted immune function, and protected the body from excessive inflammation. IgG is used for the prevention, treatment, detection, and neutralization of pathogens without closely activating the host’s own immune system (30). The concentration of IgG can reflect the immune status of animals (31). IgM plays an important role in acute infection in broilers (32). High IgM levels protect broilers from pathogens. VNE supplementation was found to increase the levels of IgG, IgA, and IgM, which can neutralize pathogens, improve the immune function of the intestinal surfaces of broilers, and ultimately boost the immune performance in chickens. IGF-1 acts on the gastrointestinal tract in several ways, including promoting the growth and survival of intestinal cells and influencing gut barrier function (33). GH promotes the proliferation of intestinal epithelial cells, enhances nutrient absorption, and influences the composition of the gut’s microbial community (34). VNE supplementation was observed to improve the levels of IGF-1 and GH in the serum, maintaining intestinal cell proliferation and promoting growth performance in broilers.

The diverse microbial community in the chicken gastrointestinal tract plays a crucial role in maintaining health and performance, with bacterial stability supporting nutrient digestion, absorption, and immune defense. Previous research has demonstrated that flavonoid supplementation contributes to balancing the intestinal flora in animals by promoting the proliferation of beneficial bacteria and reducing the presence of pathogenic bacteria (35). Consistent with this, our study found that VNE supplementation alters the composition and structure of the intestinal microbiota in broilers by increasing the abundance of beneficial bacteria, such as Bacteroides, Rikenellaceae Rc9 gut_group, and Christensenellaceae_R-7_group. Higher Bacteroides abundance has been linked to improved weight gain and feed conversion ratios in broilers, which may be attributed to efficient nutrient extraction and reduced metabolic waste (36). In chickens, these metabolic functions are critical for efficient nutrient absorption and energy utilization, directly impacting feed conversion efficiency (37). Our study also indicated that while the dominant microbial families were found to be consistent in both microbiomes, significant bacterial biomarkers emerged upon closer examination of the less abundant families. Several beneficial microbes identified in the VNE LEfSe analysis, such as the *Coprobacter fastidiosus*, Christensenellaceae R-7 group, Clostridia vadinBB60, Faecalicoccus_ pleomorphus, Clostridia UCG-014, and *Anaerofilum*, were considered important biomarkers (with a higher LDA score) in the cecal microbiota of the high group. *Coprobacter fastidiosus* produces short-chain fatty acids, such as propionic acid and acetic acid, which serve as an energy source for the host. The Christensenellaceae R7 group is associated with healthy animals and has been linked to improved feed efficiency in both chickens and pigs, by regulating energy balance and modulating hormones linked to fat storage and insulin resistance (38, 39). The role of the Clostridiales vadin BB60 group in metabolism is mostly unknown. However, some studies have suggested that these bacteria have a potential role in establishing healthy cecal flora in broiler chicks, in modulating the immune system, and in the production of SCFA (40, 41). Clostridia UCG-014 modulate the gut microbiota, improving microbial metabolites and gut barrier function through the activation of the aryl hydrocarbon receptor to upregulate epithelial tight junction proteins (42). *Faecalicoccus pleomorphus* breaks down plant-derived complex carbohydrates into simpler molecules, making them available for absorption by other bacteria or the host (43). The genus *Anaerofilum* is found in the gut microbiota, where it helps break down nutrients, produce vitamins, and support immune system development (44, 45). Pairwise correlation analysis indicated that harmful bacteria positively correlate with pro-inflammatory cytokines and pro-oxidant enzymes, while negatively correlating with anti-inflammatory cytokines, blood biochemistry parameters, and antioxidant enzymes. Previous studies have suggested that flavonoid supplementation could help prevent immune system decline in broilers (46). The gut microbiota interacts with the host’s immune system, modulating cytokine expression and affecting the severity of viral and bacterial infections. Flavonoids can regulate the gut microbiota, alleviating intestinal inflammatory injury by reducing oxidative stress and intracellular bacterial crosstalk, and enhancing antimicrobial peptides (9, 47, 48); in addition, flavonoids have been widely reported to be related to developmental states (49, 50). The modulation of intestinal metabolism can be considered another important factor affected by the intestinal microbiome (51). Overall, comparative analyses demonstrated that these key microbial groups may improve the microbial community environment by inhibiting the proliferation of harmful bacteria and promoting probiotic growth, which highlights their potential importance for VNE in both the development and absorption of the broilers.

Significant changes in metabolic pathways were identified using PICRUSt2 as opposed to Tax4Fun2. The observed enrichment of pivotal microbial metabolic pathways—specifically pyrimidine, carbohydrate, and lipid metabolism—in the high-dose VNE group provides compelling, multi-faceted mechanistic insights into improved growth performance. This systemic enhancement suggests that VNE supplementation does not merely modulate the structure of the microbial community but fundamentally amplifies its functional capacity. The upregulation of carbohydrate and lipid metabolic pathways is particularly significant, as it points to more efficient microbial fermentation of dietary components that are otherwise indigestible by the host (52). Collectively, these VNE-induced functional shifts in cecal microbiome function create a synergistic effect: a more metabolically active and stable microbial ecosystem that enhances host energy harvest, improves nutrient utilization, and ultimately results in the superior growth metrics observed in these broilers. This alignment between specific microbial metabolic functions and host zootechnical parameters significantly strengthens the premise that the cecal microbiota is a key mediator of VNE’s efficacy as a natural growth promoter.

In conclusion, the present study demonstrated that dietary VNE supplementation at 1.3–3.9 g/kg has beneficial effects on growth performance and regulates host immune function, antioxidant capacity, and serum biochemistry. It also modulates the intestinal morphology and gut microbiome of broiler chickens. The incorporation of plant extracts as feed additives is limited by the need to standardize bioactive compounds to ensure stability during feed processing and bioavailability in the animals, and to manage their variable efficacy and potential palatability issues across different species and diets. Overall, this study provides compelling evidence that VNE holds significant promise as an effective alternative to antibiotics for enhancing gut health and improving the production performance of broilers. In future research, attention should be given to the active substances and their material basis in VNE, along with their growth-promoting effects and related auspicious studies in larger populations.

## Data Availability

The original contributions presented in the study are included in the article/supplementary material, further inquiries can be directed to the corresponding author.

## Footnotes

1
https://www.majorbio.com

## References

[1] GrŽinić G Piotrowicz-Cieślak A Klimkowicz-Pawlas A Górny RL Ławniczek-Wałczyk A Piechowicz L . Intensive poultry farming: a review of the impact on the environment and human health. Sci Total Environ. (2023) 858:160014. doi: 10.1016/j.scitotenv.2022.160014, PMID: 36368402

[2] Ashour EA Aldhalmi AK Kamal M Salem SS Mahgoub SA Alqhtani AH . The efficacy of artichoke leaf extract conjugated with organic zinc nanoparticles on growth, carcass traits and blood biochemical parameters of broilers. Poult Sci. (2025) 104:104521. doi: 10.1016/j.psj.2024.104521, PMID: 39693956 PMC11720609

[3] Phillips CJC Hosseintabar-Ghasemabad B Gorlov IF Slozhenkina MI Mosolov AA Seidavi A. Immunomodulatory effects of natural feed additives for meat chickens. Life. (2023) 13:1287. doi: 10.3390/life13061287, PMID: 37374069 PMC10301336

[4] Ramesh Bahadur B Keshav B Sandesh P Deepak S Xiao Y Bidur P . Sustainable poultry farming practices: a critical review of current strategies and future prospects. Poult Sci. (2024) 103:104295. doi: 10.1016/j.psj.2024.104295 39312848 PMC11447413

[5] El-Sabrout K Ahmad S Buonaiuto G. Phytogenics, fermented ingredients, bee products, insect additives, and byproducts as promising dietary supplements for poultry. Ann Anim Sci. (2025). doi: 10.2478/aoas-2025-0049

[6] Kamal M Aldhalmi AK Abd El-Hack ME Elsherbeni AI Youssef IM Hussein S . Enhancing the feed efficiency of crop residues in ruminants–a comprehensive review. Ann Anim Sci. (2025) 25:529–45. doi: 10.2478/aoas-2024-0081

[7] El-Hack A Mohamed E Aldhalmi AK Kamal M Khafaga AF Moustafa M . Flavonoids as a phytobiotic agent: sources, classifications, biological benefits, and useful impacts on broilers and layers. Phytochem Rev. (2025):1–27. doi: 10.1007/s11101-025-10129-2

[8] Huang P Cheng P Sun M Liu X Qing Z Liu Y . Systemic review of *Macleaya cordata*: genetics, biosynthesis of active ingredients and functions. Med Plant Biol. (2024) 3:01–14. doi: 10.48130/mpb-0024-0019

[9] Zhang S Kim IH. Effect of quercetin (flavonoid) supplementation on growth performance, meat stability, and immunological response in broiler chickens. Livest Sci. (2020) 242:104286. doi: 10.1016/j.livsci.2020.104286

[10] Righi F Pitino R Manuelian CL Simoni M Quarantelli A De Marchi M . Plant feed additives as natural alternatives to the use of synthetic antioxidant vitamins on poultry performances, health, and oxidative status: a review of the literature in the last 20 years. Antioxidants. (2021) 10:659. doi: 10.3390/antiox10050659, PMID: 33922786 PMC8146777

[11] Zhu S Shen Y Zhang Q. Comparison in chemical constituents and anti-inflammatory and analgesic effects of different medicinal parts from a *Vitex negundo* L. var. cannabifolia. Fitoterapia. (2025) 185:106693. doi: 10.1016/j.fitote.2025.106693, PMID: 40532984

[12] Huang M Zhang Y Xu S Xu W Chu K Xu W . Identification and quantification of phenolic compounds in *Vitex negundo* L. var. cannabifolia (Siebold et Zucc.) hand.-Mazz. Using liquid chromatography combined with quadrupole time-of-flight and triple quadrupole mass spectrometers. J Pharm Biomed Anal. (2015) 108:11–20. doi: 10.1016/j.jpba.2015.01.049, PMID: 25703235

[13] Zeb MA Yang P-Y Bi D-W Tu W-C Li X-L Zhang R-H . Chemical constituents from *Vitex negundo* var. *cannabifolia* (Sieb. Et Zucc.) hand.-Mazz. (Lamiaceae) and their chemotaxonomic significance. Biochem Syst Ecol. (2023) 111:104719. doi: 10.1016/j.bse.2023.104719

[14] Li Y L S Tang X Yuan M Wei Q Lou H . Study on optimization of extraction process and antibacterial activity to *Clostridium perfringens* of total flavonoids from *Vitex negundo* L.var. *cannabifolia* (Sieb.et Zucc.) Hand.-Mazz. China Anim Husb Vet Med. (2022) 49:2406–13. doi: 10.16431/j.cnki.1671-7236.2022.06.040

[15] Yuan MLY He J Tang X Ba X Zhao Y Tian Y . Prevention effect of *Vitex negundo* L. var. cannabifolia (Sieb.et Zucc.) Hand.-Mazz. Extract on necrotic enteritis in broilers. China Anim Husb Vet Med. (2025) 52:2736–49. Available at: https://lib.cqvip.com/Qikan/Article/Detail?id=7201185901

[16] Cuchillo-Hilario M Fournier-Ramírez MI Díaz Martínez M Montaño Benavides S Calvo-Carrillo MC Carrillo Domínguez S . Animal food products to support human nutrition and to boost human health: the potential of feedstuffs resources and their metabolites as health-promoters. Meta. (2024) 14:496. doi: 10.3390/metabo14090496, PMID: 39330503 PMC11434278

[17] Al-Garadi MA Alhotan RA Hussein EO Qaid MM Suliman GM Al-Badwi MA . Effects of a natural phytogenic feed additive on broiler performance, carcass traits, and gut health under diets with optimal and reduced energy and amino acid density. Poult Sci. (2025) 104:105014. doi: 10.1016/j.psj.2025.105014, PMID: 40102172 PMC12540259

[18] Xu C Wei F Yang X Feng Y Liu D Hu Y. *Lactobacillus salivarius* CML352 isolated from Chinese local breed chicken modulates the gut microbiota and improves intestinal health and egg quality in late-phase laying hens. Microorganisms. (2022) 10:726. doi: 10.3390/microorganisms10040726, PMID: 35456777 PMC9029475

[19] Perera WNU Ravindran V. Role of feed additives in poultry nutrition: historical, current and future perspectives. Anim Feed Sci Technol. (2025) 326:116371. doi: 10.1016/j.anifeedsci.2025.116371

[20] Mousa MA Asman AS Ali RM Sayed RK Majrashi KA Fakiha KG . Impacts of dietary lysine and crude protein on performance, hepatic and renal functions, biochemical parameters, and histomorphology of small intestine, liver, and kidney in broiler chickens. Vet Sci. (2023) 10:98. doi: 10.3390/vetsci10020098, PMID: 36851402 PMC9965792

[21] Kalas MA Chavez L Leon M Taweesedt PT Surani S. Abnormal liver enzymes: a review for clinicians. World J Hepatol. (2021) 13:1688–98. doi: 10.4254/wjh.v13.i11.1688, PMID: 34904038 PMC8637680

[22] Chen J Dan L Tu X Sun Y Deng M Chen X . Metabolic dysfunction-associated fatty liver disease and liver function markers are associated with Crohn’s disease but not ulcerative colitis: a prospective cohort study. Hepatol Int. (2023) 17:202–14. doi: 10.1007/s12072-022-10424-6, PMID: 36194337 PMC9895026

[23] Ye W Wu W Jiang L Yuan C Huang Y Chen Z . Effects of dietary phytosterols or phytosterol esters supplementation on growth performance, biochemical blood indices and intestinal flora of C57BL/6 mice. PLoS One. (2024) 19:e0297788. doi: 10.1371/journal.pone.0297788, PMID: 38743661 PMC11093361

[24] Dakal TC Xiao F Bhusal CK Sabapathy PC Segal R Chen J . Lipids dysregulation in diseases: core concepts, targets and treatment strategies. Lipids Health Dis. (2025) 24:61. doi: 10.1186/s12944-024-02425-1, PMID: 39984909 PMC11843775

[25] Liu P Yu S Liu J Zhou Y Cao R Zhou Y . Effects of Lactobacillus on hyperlipidemia in high-fat diet-induced mouse model. Arch Med Sci. (2020) 19:792. doi: 10.3389/fnut.2024.1428577 37313183 PMC10259388

[26] Abdelli N Solà-Oriol D Pérez JF. Phytogenic feed additives in poultry: achievements, prospective and challenges. Animals. (2021) 11:3471. doi: 10.3390/ani11123471, PMID: 34944248 PMC8698016

[27] Wang Q Zhan X Wang B Wang F Zhou Y Xu S . Modified montmorillonite improved growth performance of broilers by modulating intestinal microbiota and enhancing intestinal barriers, anti-inflammatory response, and antioxidative capacity. Antioxidants. (2022) 11:1799. doi: 10.3390/antiox11091799, PMID: 36139873 PMC9495330

[28] Zhou N Tian Y Liu W Tu B Gu T Xu W . Effects of quercetin and coated sodium butyrate dietary supplementation in diquat-challenged pullets. Anim Biosci. (2022) 35:1434–43. doi: 10.5713/ab.21.0493, PMID: 35240016 PMC9449397

[29] Kumar H Dhalaria R Guleria S Cimler R Choudhary R Dhanjal DS . To exploring the role of probiotics, plant-based fermented products, and paraprobiotics as anti-inflammatory agents in promoting human health. J Agric Food Res. (2023) 14:100896. doi: 10.1016/j.jafr.2023.100896

[30] Zhang B Chen G Zhang H Lan J Yang C. Effects of rhamnolipids on growth performance and intestinal health parameters in Linnan yellow broilers. Poult Sci. (2021) 100:810–9. doi: 10.1016/j.psj.2020.10.041, PMID: 33518135 PMC7858087

[31] Fagarasan S Honjo T. Regulation of IgA synthesis at mucosal surfaces. Curr Opin Immunol. (2004) 16:277–83. doi: 10.1016/j.coi.2004.03.005, PMID: 15134775

[32] Wang H Coligan JE Morse HC 3rd. Emerging functions of natural IgM and its fc receptor FCMR in immune homeostasis. Front Immunol. (2016) 7:99. doi: 10.3389/fimmu.2016.00099, PMID: 27014278 PMC4791374

[33] Kuemmerle JF. Insulin-like growth factors in the gastrointestinal tract and liver. Endocrinol Metab Clin N Am. (2012) 41:409–23. doi: 10.1016/j.ecl.2012.04.018 PMC3372868 22682638

[34] Jensen EA Young JA Jackson Z Busken J List EO Carroll RK . Growth hormone deficiency and excess alter the gut microbiome in adult male mice. Endocrinology. (2020) 161:bqaa026. doi: 10.1210/endocr/bqaa026, PMID: 32100023 PMC7341558

[35] Xiong HH Lin SY Chen LL Ouyang KH Wang WJ. The interaction between flavonoids and intestinal microbes: a review. Foods. (2023) 12:320. doi: 10.3390/foods12020320, PMID: 36673411 PMC9857828

[36] Fan Y Ju T Bhardwaj T Korver DR Willing BP. Week-old chicks with high Bacteroides abundance have increased short-chain fatty acids and reduced markers of gut inflammation. Microbiol Spectr. (2023) 11:e0361622. doi: 10.1128/spectrum.03616-22, PMID: 36719194 PMC10100795

[37] Clavijo V Flórez MJV. The gastrointestinal microbiome and its association with the control of pathogens in broiler chicken production: a review. Poult Sci. (2018) 97:1006–21. doi: 10.3382/ps/pex359, PMID: 29253263 PMC5850219

[38] Liao SF Ji F Fan P Denryter K. Swine gastrointestinal microbiota and the effects of dietary amino acids on its composition and metabolism. Int J Mol Sci. (2024) 25:1237. doi: 10.3390/ijms25021237, PMID: 38279233 PMC10816286

[39] Xu C Jiang H Feng L-J Jiang M-Z Wang Y-L Liu S-J. *Christensenella minuta* interacts with multiple gut bacteria. Front Microbiol. (2024) 15:1301073 38440147 10.3389/fmicb.2024.1301073 PMC10910051

[40] Hao G Li P Huang J Cui K Liang L Lin F . Research note: therapeutic effect of a *Salmonella* phage combination on chicks infected with *Salmonella Typhimurium*. Poult Sci. (2023) 102:102715. doi: 10.1016/j.psj.2023.102715, PMID: 37209652 PMC10208875

[41] Paneru D Tellez-Isaias G Bottje WG Asiamah E Abdel-Wareth AAA Salahuddin M . Immune modulation and cecal microbiome changes in broilers fed with fenugreek seeds and Bacillus-based probiotics. Poult Sci. (2024) 103:104130. doi: 10.1016/j.psj.2024.104130, PMID: 39121644 PMC11364116

[42] Ratto D Roda E Romeo M Venuti MT Desiderio A Lupo G . The many ages of microbiome-gut-brain axis. Nutrients. (2022) 14:2937. doi: 10.3390/nu14142937, PMID: 35889894 PMC9319041

[43] Vivek S Shen YS Guan W Onyeaghala G Oyenuga M Staley C . Association between circulating T cells and the gut microbiome in healthy individuals: findings from a pilot study. Int J Mol Sci. (2024) 25:6831. doi: 10.3390/ijms25136831, PMID: 38999941 PMC11241708

[44] Von Martels JZH Sadaghian Sadabad M Bourgonje AR Blokzijl T Dijkstra G Faber KN . The role of gut microbiota in health and disease: in vitro modeling of host-microbe interactions at the aerobe-anaerobe interphase of the human gut. Anaerobe. (2017) 44:3–12. doi: 10.1016/j.anaerobe.2017.01.001, PMID: 28062270

[45] Zhi W Tang K Yang J Yang T Chen R Huang J . Research on the gut microbiota of Hainan black goat. Animals. (2022) 12:3129. doi: 10.3390/ani12223129, PMID: 36428357 PMC9686789

[46] Buiatte V Dominguez D Lesko T Jenkins M Chopra S Lorenzoni AG. Inclusion of high-flavonoid corn in the diet of broiler chickens as a potential approach for the control of necrotic enteritis. Poult Sci. (2022) 101:101796. doi: 10.1016/j.psj.2022.101796, PMID: 35364456 PMC8968645

[47] Duarte-Mata DI Salinas-Carmona MC. Antimicrobial peptides´ immune modulation role in intracellular bacterial infection. Front Immunol. (2023) 14:1119574. doi: 10.3389/fimmu.2023.1119574, PMID: 37056758 PMC10086130

[48] Oke O Akosile O Oni A Opowoye I Ishola C Adebiyi J . Oxidative stress in poultry production. Poult Sci. (2024) 103:104003. doi: 10.1016/j.psj.2024.104003, PMID: 39084145 PMC11341942

[49] Insawake K Songserm T Songserm O Theapparat Y Adeyemi KD Rassmidatta K . Flavonoids, isoquinoline alkaloids, and their combinations affect growth performance, inflammatory status, and gut microbiome of broilers under high stocking density and heat stress. Animals. (2025) 15:71. doi: 10.3390/ani15010071, PMID: 39795014 PMC11718826

[50] Liu C Huang H Chen Y Zhou Y Meng T Tan B . Dietary supplementation with mulberry leaf flavonoids and carnosic acid complex enhances the growth performance and antioxidant capacity via regulating the p38 Mapk/Nrf2 pathway. Front Nutr. (2024) 11:11–2024. doi: 10.3389/fnut.2024.1428577, PMID: 39139650 PMC11319276

[51] Sharifuzzaman M Mun H-S Ampode KMB Lagua EB Park H-R Kim Y-H . Optimizing broiler growth, health, and meat quality with citric acid-assessing the optimal dose and environmental impact: citric acid in broiler health and production. Poult Sci. (2025) 104:104668. doi: 10.1016/j.psj.2024.104668, PMID: 39705837 PMC11728898

[52] Ramasinghe C Bordiga M Xu B. A comprehensive review of the triangular relationship among diet, gut microbiota, and aging. Int J Mol Sci. (2025) 26:8785. doi: 10.3390/ijms26188785, PMID: 41009354 PMC12469625

